# Anæsthesia and Anæthetics

**Published:** 1888-03

**Authors:** 


### ANÆSTHESIA AND ANÆSTHETICS.

We have received many letters from subscribers asking for an article, or a series of articles, upon anæsthetics and their mode of administration, to be written for the benefit of those who have had no special schooling in this branch of practice—plain, practical, and with as few technicalities as possible. We have determined to comply with these requests to the best of our ability, and to present our ideas as opportunity offers. It should be premised that, although we must speak didactically, we do not pretend to be authoritative, and what we say must be taken for what it is worth. Some years since we made a considerable study of the subject, and performed many experiments upon the lower animals, and it is upon this series of observations that we shall speak, drawing conclusions from our own studies, rather than from the writings of others. We shall not pretend to follow the usually accepted authorities, and if we antagonize them our readers must judge between us. Only do not let it be thought that we despise or undervalue the wisdom of the many who have written upon the subject. We simply prefer to express our own opinions, even though they may be crude and illogical, rather than to attempt to repeat what has been better expressed before.

To comprehend the effects of any drug upon nervous tissues, and what, for want of a better term, we may call the nervous currents, it is necessary that we enquire a little into the character of nervous force. In a paper read before the American Dental Association, at its meeting in Cincinnati in 1882, we gave our views at length, and we would like to refer the reader to the transactions of the society for that year for a full exposition of what we believe to be the origin and character of nervous force. At present we will only attempt briefly to summarize our views. For a full comprehension of the matter the student should be acquainted with the later theories concerning the nature of force, and to this end he should read "The Correlation and Conservation of Forces," by Profs. Grove, Helmholtz and Liebig, and Drs. Faraday, Mayer and Carpenter, edited by Prof. Youmans and published by D. Appleton & Co., New York. Some of the views therein expressed have been modified by later observations, but the book in the main stands as the best and most consistent system of the philosophy of force which has been presented.

All the different manifestations of force are mutually convertible the one into the other, and are practically a unit. Heat, light, electricity, and what is denominated chemical activity, are but the different modes by which force is manifested. Commencing with the simple battery cell, we see the chemical activity there exhibited made manifest as electricity. If a closed circuit be made by means of conducting wires in which a piece of thin platinum is inserted, the electric current is changed in character, and is manifested as heat. There is no change, save in the manner of its exhibition, but the invisible manifestation of the current is now made sensible to the eye, and we give it another name. It is the same force, but it has assumed a new form. If there be a total break in the circuit instead of a mere resistance, the same electricity is made manifest in yet another manner, and we call it light.

Here we have four different forms under which the same force appears, each mutually resolvable into the other, for heat and light may in turn induce an electric current or chemical action. We are then warranted in pronouncing them essentially identical, differing only in their mode of manifestation. The great error in the earlier philosophies arose from considering them as entities, each distinct from the other, and hence come the terms "positive" and "negative" electricity; "sensible" and "latent" heat, etc. We must take care to avoid this mistake and to look upon them as simply manifestations of an imponderable force, which is the active agent in producing all the changes in passive matter. We have no knowledge in this world of anything but the inert matter which constitutes all created things, and the mysterious force which is only made sensible by its action upon matter.

Pursuing this train of reasoning, and looking at the matter from a purely physiological and material standpoint, and without any reference to a spiritual or immaterial principle which may form another part of our existence, we are able to view our bodies as composed of inert matter, which is made physically active by some exhibition of the unit force. This physiological activity, this manifestation of force, is, we believe, but another exhibition of that great force which acts upon all matter, and it is denominated nervous force. As by the molecular changes within the battery cell, under the influence of chemical affinity, we see an evolution of that form of force which we call electricity, so within the active animal body the molecular changes which take place under the influence of what we call digestion, result in the evolution of nervous force.

Nor does this manifestation essentially differ in character from the other forms of the unit force. It is at times made manifest as heat, and even as electricity, Light, heat and electricity are, under certain circumstances, converted into nervous force, and *vice versa.* I might cite a great many instances of this, but the thoughtful student of physiology will readily recall them. Many of the diseases to which we are subject are due to the mysterious conversion of nerve force into heat, etc. There is an order of fishes which, by peculiar nervous organs, has the power at will to change nerve force into electricity, and there are insects which convert it into light, in the same manner.

We shall then consider nerve or vital force as essentially identical with the other manifestations of force, and nervous and ganglionic tissue as that whose peculiar office it is to convert other forms of force into nervous force, and to convey or conduct its undulations or influence to the different organs, or aggregations of animal matter.

(TO BE CONTINUED.)

